# Laryngeal *Mycobacterium bovis*: A Unique Cause of Airway Compromise in a 27-Year-Old Male With Down Syndrome

**DOI:** 10.1155/crot/6485801

**Published:** 2025-09-11

**Authors:** Morgan Davis Mills, Michael Oca, Michelle Don, Andrew M. Vahabzadeh-Hagh

**Affiliations:** ^1^Department of Otolaryngology, Head and Neck Surgery, University of California San Diego, San Diego, California, USA; ^2^School of Medicine, University of California, San Diego, California, USA; ^3^Department of Pathology, University of California, San Diego, California, USA

**Keywords:** granulomatous mass, laryngeal mass, laryngeal tuberculosis, *Mycobacterium tuberculosis*

## Abstract

**Introduction:** Laryngeal tuberculosis (TB) due to *Mycobacterium bovis* is an extremely rare cause of airway obstruction. This case report describes a unique instance of acute airway obstruction in an immunocompetent 27-year-old male with down syndrome caused by laryngeal *Mycobacterium bovis*, shedding light on the challenges of diagnosis and treatment.

**Case:** A 27-year-old male with trisomy 21 presented with progressive shortness of breath, productive cough, dysphonia, and dysphagia. After a failed workup for pneumonia and other conditions, imaging revealed likely epiglottitis and a right upper lung lesion. A tracheostomy was performed due to worsening airway compromise. Biopsy results confirmed granulomatous inflammation and identified *Mycobacterium bovis*, which was resistant to pyrazinamide. The patient was treated with a modified RIPE regimen and successfully decannulated 2 months later.

**Conclusion:** This case emphasizes the importance of a comprehensive diagnostic approach, including tissue biopsy and culture, in patients with airway compromise of unclear etiology. *Mycobacterium bovis*, though rare, should be considered in the differential diagnosis of laryngeal TB, especially in cases with progressive symptoms and atypical findings. Early recognition and tailored treatment are critical for favorable outcomes.

## 1. Introduction

Since the widespread use of antibiotics, laryngeal manifestations of tuberculosis (TB) are a rare entity accounting, for < 1% of pulmonary TB cases [1]. Most TB cases, including laryngeal TB, are caused by *Mycobacterium tuberculosis*, with only two cases of laryngeal TB due to *Mycobacterium bovis* (*M. bovis*) reported in the literature [2, 3]. Details from those reports are limited, underscoring the exceptional rarity of this presentation. Here, we present a unique case of airway obstruction secondary to laryngeal *M. bovis* in an immunocompetent patient.

## 2. Case

A 27-year-old male with trisomy 21 and intellectual disability presented to a tertiary medical center with 6 months of slowly progressive shortness of breath, productive cough, stridor, and night sweats. The patient's mother reported progressive voice loss over 2 months, dysphagia, and choking on all consistencies. The patient had undergone evaluations by nine different physicians across multiple specialties (ENT, pulmonology, GI, allergy) in Mexico and the USA and trialed numerous antibiotic courses for presumed pneumonia, antihistamines, inhalers, and glucocorticoids without significant improvement. There was no history of immunodeficiency, significant travel aside from brief trips to Mexico, or diabetes. Random blood glucose values were 95 mg/dL during admission and 97 mg/dL 1 year prior; no HbA1c was obtained. Social history revealed that the patient lived with his mother in El Centro, frequently visited an aunt in Mexicali, and consumed unpasteurized dairy products from Mexico. His mother denied known TB contacts but reported exposure to areas with a high prevalence of homelessness and illness.

**Figure 1:**
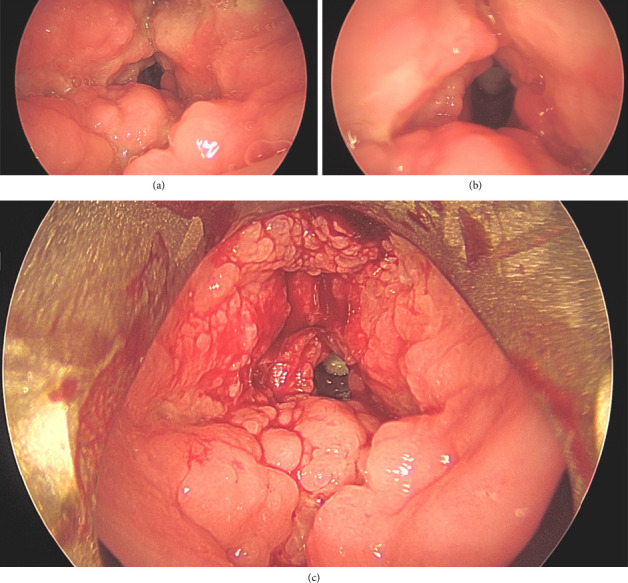
Intraoperative findings from direct laryngoscopy. (a) Diffuse pseudotumor appearance of bilateral false vocal folds with difficult identification of normal laryngeal anatomy. (b) Abnormal appearance of bilateral true vocal folds. (c) Supraglottis and glottis after biopsies performed. Additional lesions were seen on laryngeal surface of the epiglottis.

A computed tomography (CT) scan from an outside hospital suggested epiglottitis and a right upper lung cavitary lesion, prompting transfer to UCSD. On arrival, he had inspiratory stridor, dysphonia, and dyspnea. Flexible fiberoptic laryngoscopy (FFL) showed a boggy epiglottis with moderate edema, copious white frothy secretions, and exophytic supraglottic masses obstructing the airway. An awake tracheostomy was performed for airway stabilization, followed by direct laryngoscopy and bronchoscopy with biopsies. Intraoperative findings revealed diffuse firm, pseudo-tumor-like ulceroproliferative lesions involving the epiglottis, false vocal folds, ventricle, arytenoids, and true vocal folds (Figure 1). Flexible bronchoscopy demonstrated a normal tracheobronchial tree without any evidence of lesions or infiltrative mass.

**Figure 2:**
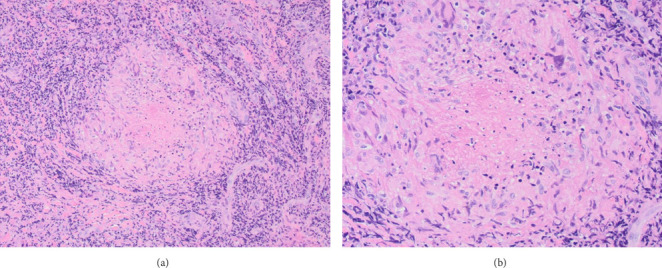
H&E staining of the supraglottic biopsy showing a granuloma with central granular caseation surrounded by epithelioid histiocytes and giant cells at 10x objective (a) and 20x objective (b).

**Figure 3:**
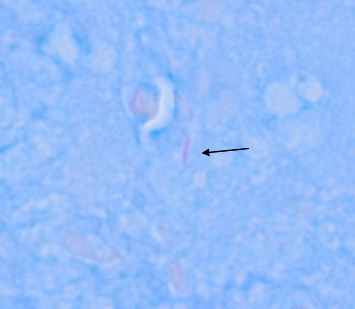
Acid-fast staining of the supraglottic biopsy shows a single rare positive organism at 100x magnification.

Histopathological examination showed granulomatous inflammation with caseating necrosis, epithelioid histiocytes, chronic inflammatory cells, and giant cells (Figure 2). Acid-fast bacilli (AFB) staining identified a single bacillus, confirming TB infection (Figure 3). On admission, records from the outside hospital documented a positive MTB-RIF GeneXpert result, whereas induced sputum samples obtained at our institution were smear negative, MTB PCR was negative, and the Quantiferon test was uninterpretable. HIV testing was negative.

Species identification was confirmed by the Imperial County Public Health Laboratory based on culture growth characteristics consistent with *M. bovis* and pyrazinamide resistance on susceptibility testing. Autoimmune causes such as granulomatosis with polyangiitis (GPA) were considered; however, GPA was felt less likely given the patient's epidemiologic risk factors, cavitary lung lesion, normal admission CBC, and absence of sinonasal, renal, or cutaneous involvement. Inflammatory markers and ANCA serologies were not obtained. A positive MTB-RIF GeneXpert from the outside hospital and early biopsy histopathology showing caseating granulomas with a rare AFB further supported a mycobacterial etiology over autoimmune vasculitis.

Empirical rifampin, isoniazid, pyrazinamide, and ethambutol (RIPE) therapy was initiated. One month later, biopsy and sputum cultures confirmed *M. bovis*, resistant to pyrazinamide. The patient was transitioned to rifampin, isoniazid, and ethambutol with vitamin B6. He was admitted to a negative pressure room under airborne precautions for the entire hospital stay, with isolation discontinued only at discharge per infectious disease recommendations. He was discharged to a short-term nursing facility and successfully decannulated 2 months post-tracheostomy.

## 3. Discussion

In the United States, *M. bovis* accounts for < 2% of TB cases [4], though its prevalence is higher in regions like Southern California, particularly among children and immunocompromised patients [5–7]. Infection is primarily acquired through ingestion of unpasteurized dairy products, but human-to-human transmission via aerosols or droplets can occur. *M. bovis* predominantly affects the lungs but can involve extrapulmonary sites, including the larynx. Laryngeal TB, though rare, presents with dysphonia in > 70% of cases [8, 9], along with stridor, dyspnea, odynophagia, and dysphagia. TB may involve any of the laryngeal subsites and manifest as edema, hyperemia, nodularity, ulceroproliferative lesions, exophytic masses, or complete obliteration of anatomic landmarks on laryngoscopy. Biopsy is essential for diagnosis, revealing caseating granulomas and AFB on histopathology. Tissue biopsy is also indicated to rule out squamous cell carcinoma (SCC) of the larynx which may be clinically indistinguishable as well as histopathologically similar in appearance due to the presence of pseudoepitheliomatous hyperplasia [8]. Biopsies should be taken from all suspicious lesions and various subsites, as TB and SCC may coexist, particularly in patients at risk for SCC (e.g., heavy alcohol and tobacco use).

Treatment requires culture-sensitive anti-TB medications. *M. bovis* is intrinsically resistant to pyrazinamide, requiring tailored regimens [10, 11]. In cases of airway compromise, tracheostomy may be necessary until medical therapy reduces lesion burden. Surgical intervention is rarely needed, as most laryngeal TB lesions respond to treatment within 2–3 months [1]. Compared to *M. tuberculosis*, *M. bovis* TB carries a worse prognosis, with reported mortality rates of 9% versus 5% in the U.S. and 20% versus 4% in the Netherlands [12, 13]. Early recognition and treatment are critical, particularly in patients with atypical presentations or unexplained airway symptoms.

This report underscores the critical importance of considering *M. bovis* as a rare but significant cause of laryngeal TB, particularly in cases of unexplained airway obstruction. Timely airway management and histopathological confirmation remain paramount for guiding effective treatment. Given the intrinsic resistance of *M. bovis* to pyrazinamide and its potential for severe disease progression, early initiation of an appropriate anti-TB regimen is essential. Clinicians should maintain a high index of suspicion in endemic regions and among patients with persistent laryngeal symptoms unresponsive to conventional therapies. This case highlights the necessity of rapid diagnostic evaluation and targeted therapeutic intervention to optimize patient outcomes.

## Data Availability

The data that support the findings of this study are available on request from the corresponding author. The data are not publicly available due to privacy or ethical restrictions.
